# MASCDB, a database of images, descriptors and microphysical properties of individual snowflakes in free fall

**DOI:** 10.1038/s41597-022-01269-7

**Published:** 2022-05-03

**Authors:** Jacopo Grazioli, Gionata Ghiggi, Anne-Claire Billault-Roux, Alexis Berne

**Affiliations:** Environmental Remote Sensing Laboratory, École Polytechnique Fédérale de Lausanne, Lausanne, Switzerland

**Keywords:** Hydrology, Cryospheric science

## Abstract

Snowfall information at the scale of individual particles is rare, difficult to gather, but fundamental for a better understanding of solid precipitation microphysics. In this article we present a dataset (with dedicated software) of *in-situ* measurements of snow particles in free fall. The dataset includes gray-scale (255 shades) images of snowflakes, co-located surface environmental measurements, a large number of geometrical and textural snowflake descriptors as well as the output of previously published retrieval algorithms. These include: hydrometeor classification, riming degree estimation, identification of melting particles, discrimination of wind-blown snow, as well as estimates of snow particle mass and volume. The measurements were collected in various locations of the Alps, Antarctica and Korea for a total of 2’555’091 snowflake images (or 851’697 image triplets). As the instrument used for data collection was a Multi-Angle Snowflake Camera (MASC), the dataset is named MASCDB. Given the large amount of snowflake images and associated descriptors, MASCDB can be exploited also by the computer vision community for the training and benchmarking of image processing systems.

## Abstract (summary)

**Table:** 

Measurement(s)	fall speed • snowfall images • snow crystals • geometrical characteristics • textural characteristics • snowfall microphysics
Technology Type(s)	multi angle snowflake camera

## Background & Summary

Snowfall is a phenomenon of extraordinary complexity across all temporal and spatial scales. At the scale of individual particles, the characteristics of a snow crystal or a snowflake (an aggregate of more individual crystals) depend in the first place on the environmental conditions upon formation and then on the environments encountered as the particle crosses the atmosphere *from cloud top to ground deposition*. Individual crystals may branch with each other and form aggregates^1,2^. They may collide with supercooled liquid water droplets that will freeze on them upon impact (riming^3,4^), they may sublimate or generate secondary ice by breaking up or splintering^5^. Measuring, describing and understanding the interactions occurring at the microphysical scale are complex tasks and important limiting factors to represent these mechanisms in numerical weather prediction models or climate models^6^. Because of computational constraints, these models are often unable to simulate processes at this scale and unavoidably adopt empirical parametrizations to get close to the observations^7^.

The research community compiled in the last half century a large inventory of snow crystal types^8^. This has been possible thanks to a double effort: years of manually-collected field observations from one side complemented by experiments in controlled laboratory conditions. Laboratory experiments have the special merit to quantitatively define the environmental factors leading to the formation of crystals with different shapes and types^9^. Although a taxonomy of individual crystals is nowadays established, much more has to be investigated about mass, density, fall speed and orientation and all the dynamical processes occurring while solid hydrometeors fall. A helping hand to the research in this field comes from the rapid development of snow particle imagers, either installed on aircraft to sample clouds and precipitation aloft^10–12^ or deployed at the ground level^13–16^. Ground-based imagers observe snowfall just before deposition and can provide information also on the fall speed, orientation and textural characteristics of falling snowflakes. The data collected with devices producing actual high resolution hydrometeor pictures, like the Multi-Angle Snowflake Camera (MASC)^15^, allow for immediate visual interpretation of the images. MASC data, for example, were used to perform hydrometeor classification^17–20^ as well as riming degree estimation of individual particles^17^ or, at the scale of a population of particles, to discriminate between snowfall and wind-blown snow^21^.

Three techniques have appeared in the literature to classify MASC images into solid hydrometeor types. In a supervised learning setting, Praz *et al*.^17^ employed expert knowledge for feature extraction and multinomial logistic regression for snowflake classification, while Hicks *et al*., 2019 and Key *et al*.^18,20^ described the use of convolutional neural networks architecture for the same purpose. On the other hand, Leinonen *et al*.^19^ presented an unsupervised approach where the expressive power of generative adversarial networks is employed to extract snowflake image characteristics without human expertise, which are then used to perform clustering using k-medoids.

MASC data have been exploited to propose three dimensional reconstruction techniques^22,23^ and have been more in general proven useful to better describe, quantify and interpret the microphysical properties of falling snow^3,24–27^.

Although the potential of MASC is indubitable, the studies listed above focused on individual field campaigns and/or on the development of specific retrievals possibly obtained using different data filtering and preprocessing techniques. This work aims to fill such gap and to provide the scientific community with a standardized large database (named MASCDB) covering at the present time ten field campaigns worldwide. The database includes MASC images, image descriptors and precomputed output of existing classification and retrieval algorithms. Raw MASC images have been treated within a homogeneous processing chain and additional effort has been devoted to add basic environmental information to all the collected measurements. Additionally, an intuitive and well documented python package has been developed to facilitate MASCDB data extraction, manipulation, exploration, visualization and to provide the possibility to extend the database with additional image descriptors and retrievals.

MASCDB is the first outcome of an international collaborative effort to promote exchange of data across the precipitation research scientific community and this precompiled dataset can be useful in several ways. First, it will allow to enhance scientists’ productivity, by focusing directly and efficiently on the improvement/development of microphysical retrievals and the analysis of snowflake characteristics, without the need to devote time to the reimplementation of preprocessing and retrieval algorithms which are already provided by this study. The large number of snowflakes available will allow to better characterize snowflake variability, while the included snowflake property estimates unlock potential to advance the accuracy of weather radar snowfall microphysical retrievals, and the robustness of microphysical schemes in weather and climate models.

The complexity of snowflake shapes in MASCDB images and their translation- and rotation-invariant semantic characteristics represents an ideal test-bed for the development and improvement of representation learning and image classification algorithms. Therefore, MASCDB also holds potential to be adopted as a benchmark dataset by the computer vision and machine learning community for the development of classifiers, as well as to test the ability of representation learning and generative algorithms to disentangle, extract, and reproduce the complex variety of snowflakes that nature can offer.

Finally, thanks to a well documented support software and to the overall compact size of the database, MASCDB could emerge as a useful tool for educational activities and exercise sessions in atmospheric and computer vision curricula.

MASCDB can be accessed on *Zenodo*^28^, while the *pymascdb* package at https://github.com/ltelab/pymascdb.

## Methods

### Multi-angle snowflake camera (MASC)

**Fig. 1:**
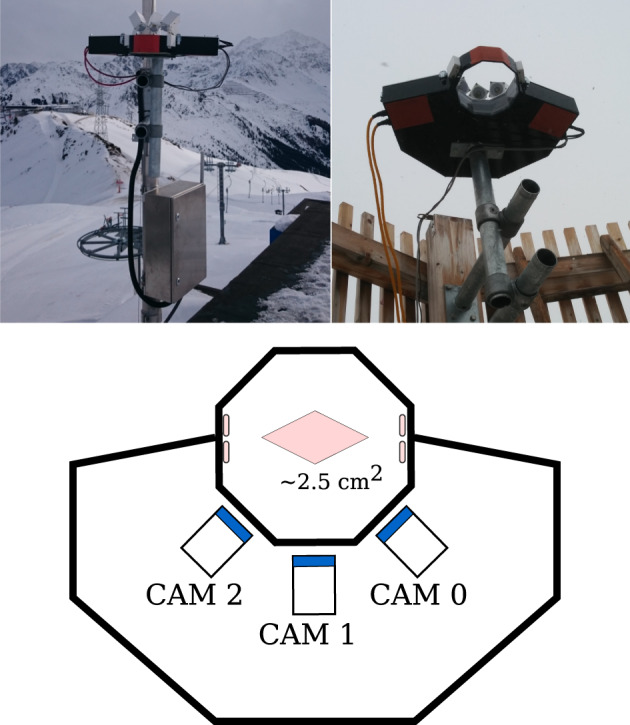
Side view, bottom view and schematics of a Multi-Angle Snowflake Camera^15^.

The Multi-Angle Snowflake Camera (MASC^15^) is an instrument able to collect a triplet of high resolution images of falling hydrometeors and to provide an estimate of their fall speed. A MASC, as illustrated in Fig. 1, is composed of three high resolution co-planar cameras with the same focal point situated at an approximate distance of 10 cm. The cameras are separated by 36° with respect to the next one such that a picture of each snowflake can be obtained simultaneously at an angle of 0° and ±36°, covering thus a total span of 72°. Two infrared (IR) emitter-detector pairs are triggering the cameras and three LED spotlights illuminate the targets. The IR arrays are separated vertically by 32 mm, providing in this way an estimate of particle fall velocity. The data processed and provided here have been collected with two identical MASC system, equipped with three 2448 × 2048 pix cameras of 12.5 mm focal length (*f*) and aperture *f*/5.6 operating with a maximum acquisition rate of about 2 Hz and an exposure time of 40 *μ*s^17^. The pixel resolution in this set up is 33.5 *μ*m. The sampling area is relatively small (nominally, according to specifications is 2.5 cm^2^) and this helps to avoid as much as possible multiple triggers of the IR detector by different particles. In calm air this is generally true, but as soon as the wind speed increases the sampling of larger snowflakes becomes less efficient^29^ and at the same time multiple triggers from smaller snowflakes or blowing snow particles can occur. In case of wind-blown snow, which is snow recirculated from the ground, several small particles may be present in the measurement area at the same time^21^. This is a known limitation and one of the reasons behind our effort to provide environmental information within our database, as detailed in the following sections.

### Data processing chain

**Fig. 2:**
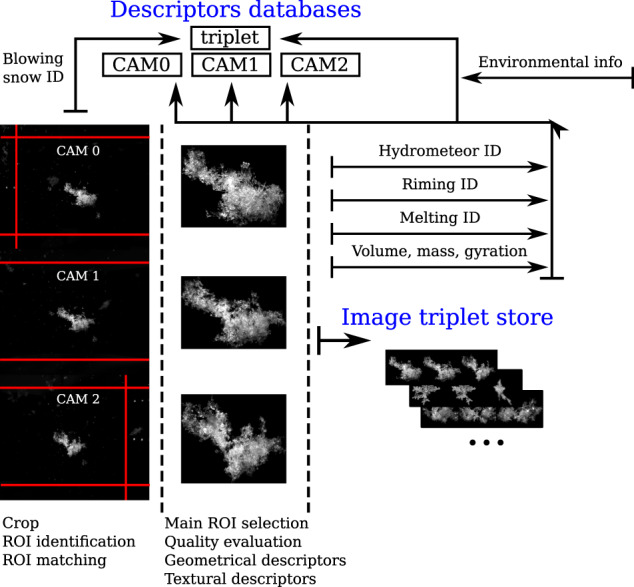
Schematic concept of data processing sequence, from raw MASC images to storage into the four descriptor databases (one for each cam plus one for the full triplet) and the store of triplet images.

Raw data consists of a triplet of 2448 × 2048 gray-scale images (see Fig. 2) with values in the range 0–255 (black-white) collected by the three cameras. As a reference, CAM0 is the leftmost camera, for an observer facing the instrument (Fig. 1). The images are at first cropped around the borders. All images are cropped at the top and at the bottom, in order to remove the image background not belonging to the measurement area. CAM0 (CAM1) images are further left- (right-) cropped as the bright LEDs of the infrared beams appear in the pictures. To reduce the background noise, pixels with values lower than 12 are set to 0.

After this preliminary treatment, the images are processed in order to identify connected regions of interest (ROIs). To this end, the greyscale images are binarized. Any hole (single 0 pixels completely surrounded by ones) in the resulting mask is filled with ones. The mask is applied to the original gray-scale image and it is used to crop and extract around every separate ROI. ROIs with an area smaller than 10 pixels are discarded as well as ROIs touching the borders of the image.

The ideal measurement concept is the following: an individual snowflake crosses the measurement area, triggers the two IR detectors, a triplet of pictures is collected and a unique ROI is found on each image. In reality, in our dataset, individual ROIs are found in an image only approximately once every eight triggers. When precipitation is intense or mixed with blowing snow, or a large number of small particles are present, multiple objects appear on each image. Consequentially, the next step involves the matching of ROIs of individual cameras into triplets that are associated to the same snowflake. This is done by comparing their average vertical position and average vertical size, as this is a common dimension among the three cameras and it should approximately match. For each ROI, the central camera is taken as a reference and matches are searched in the other cameras. A best match is found among the particles having a maximum difference in vertical position and dimension with respect to the reference that is within 50 pixels (or within 100% of the reference value if this is larger than 50 pixels). Among all the matching triplets, we decided to keep only the one that is on average the sharpest and brightest, assuming that it corresponds to the best placed particle within the sampling area. The parameter used as quality discriminant is named *AF* (Area-Focus):

1 $$AF=A\times F$$

where *A* is the area of the ROI in pixels and *F* is a focus factor defined as:

2 $$F=\frac{\bar{I}}{255}\times {\left(\frac{\bar{{R}_{I}}}{255}\right)}^{2}$$

with $\bar{I}$ the mean intensity of pixels belonging to the ROI and $\bar{{R}_{I}}$ the mean range intensity (local 3 × 3 *max*–*min* operator) of the same pixels.

The available data are further filtered prior inclusion into MASCDB in order to eliminate images with poor quality. The quality parameter *ξ* (as thoroughly described in Appendix B of^17^) is used as a discriminant. *ξ* quantifies the blurriness of the images as much as possible independently from their brightness. *ξ* values above 9 usually correspond to very sharp images, while values below 8 to very blurry ones. In previous studies, thresholds on *ξ* of 9^17^ or 10^19^ have been applied for the purpose of hydrometeor classification, which requires very sharp and high-quality images promptly recognizable by visual inspection. Some geometrical descriptors, less influenced by texture, may still be quantitatively valuable even for low-quality images and therefore we selected a more permissive threshold for our database. A triplet of ROIs is accepted if each view has *ξ* of at least 8 or alternatively if at least one view has *ξ* larger than 8.5. The last condition in particular serves a double purpose: it preserves individual good quality images in overall bad quality triplets and it allows to include in the database also low quality images potentially useful for future image classification applications. As summarized in Table 1, MASCDB currently includes 851’697 triplets, corresponding to 2’555’091 snowflake images.

**Table 1:** List of field campaigns for which data are available in the dataset.

Campaign	Start	End	*# triplets*	Ref
*Davos-2015*	2015-02-10	2016-06-19	207’194	^17^
*APRES3-2016*	2015-11-11	2016-01-29	58’836	^30,33^
*Valais-2016*	2016-12-19	2017-04-03	119’261	
*APRES3-2017*	2017-01-10	2017-07-17	328’573	^21,33^
*ICEPOP-2018*	2018-02-21	2018-03-21	25’113	^34^
*PLATO-2019*	2018-11-29	2019-02-06	760	^35^
*Davos-2019*	2019-02-22	2019-03-26	41’474	^36,88^
*Jura-2019*	2019-11-14	2020-04-30	36’394	
*POPE-2020*	2019-11-30	2020-01-06	17’353	
*ICEGENESIS-2021*	2020-12-10	2021-03-17	16’739	

Note that full information on the exact locations is available directly in the data as well as in the provided codes. When available, the column *Ref* includes references of published literature with more information about the field campaign.

**Fig. 3:**
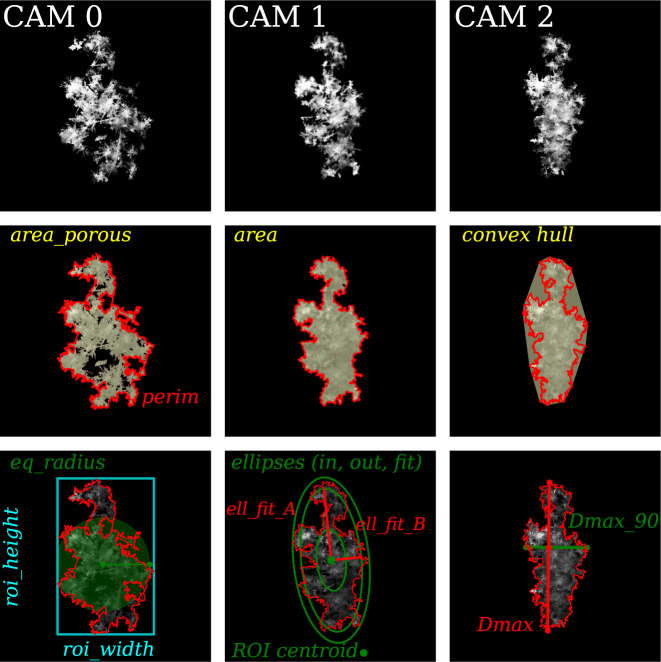
Example of a triplet of camera images. Each camera view is used to illustrate a few geometrical descriptors, among the ones available in the dataset and listed in Supplementary Table 4.

As it will be detailed in the following section (Data Records), MASCDB includes the actual gray-scale images of the ROIs of each triplet, in addition to a set of precomputed geometrical and textural parameters as well as a set of precomputed retrievals from published algorithms (Supplementary Tables 3 and 4). The geometrical and textural descriptors of each ROI are in large part the same ones as derived in Praz *et al*.^17^. A few visual examples are depicted in Fig. 3 and a complete list with relevant references and description is summarized in Supplementary Table 4.

### Data from retrieval algorithms

The precomputed products provided in MASCDB and listed in the entries of Supplementary Tables 3 and 4 are briefly describe here.

#### Fall speed

The fall speed of the hydrometeors observed by the MASC is estimated by the difference in time between the trigger of the upper and lower IR emitter-detector pairs. This attribute is available in MASCDB with name *flake_fallspeed*. It has been shown in past research that wind and turbulence affect this measurement^3,29^; these studies suggest that, in order to maximize the reliability of the estimate, the MASC should be sheltered from the wind with a double fence and wind speed should be below 5 ms^−1^. As a reference, in MASCDB, about 35% of the data are associated to winds below this threshold. If we consider only the two field campaigns during which a double fence was available, this percentage drops to 11.5%.

#### Hydrometeor classification

**Fig. 4:**
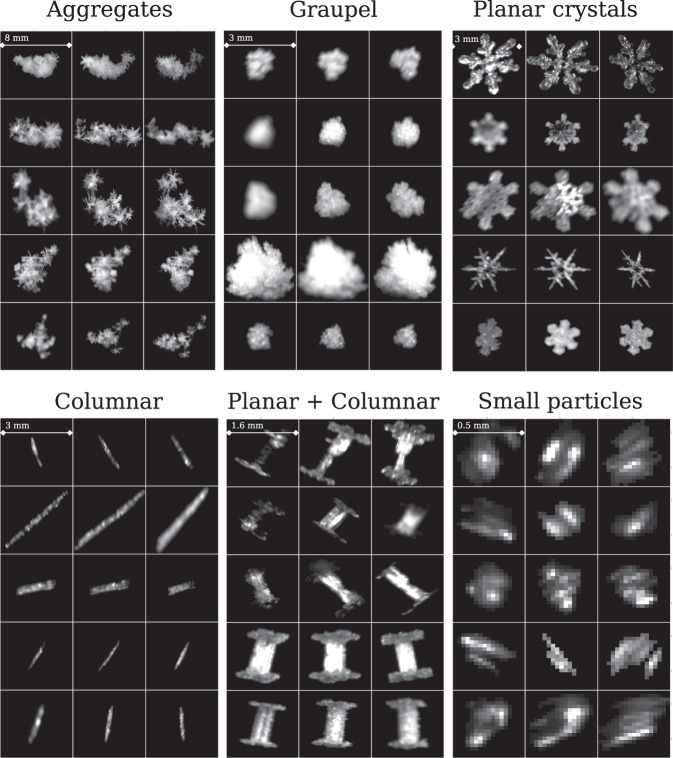
Example of triplets included in MASCDB and stratified according to the available output of the hydrometeor classification method of *Praz et al*.^17^. Triplets are selected to be of good quality (average *ξ* above 9) and with high classification probability values.

The output class of the hydrometeor classification of Praz *et al*.^17^ is provided together with the classification probability of the assigned class. The output is provided both individually for each ROI in a triplet and as a unique value considering the triplet images altogether. The classification method discriminates between: small ice particles, aggregates, planar crystals (sectored plates, plates, stellar crystals/dendrites), columnar crystals (needles, columns), graupel and combination of planar crystals and columns (for example capped columns). It must be noted that the method does not discriminate some important ice-phase hydrometeors like hail, bullets (probably classified as columnar crystals) or combination of bullets as bullet rosettes. Hydrometeor classification outputs are stored in MASCDB in attributes starting with *snowflake_class_*. An example of good quality and high probability image triplets in the database belonging to the six hydrometeor classes is illustrated in Fig. 4. These correspond to good cases where the overall classification probability is very high: the hydrometeor types are clearly recognizable in every image of the triplet. It can also happen that only one or two cameras provide high-quality classification or, because of the different viewpoints, the classification output differs among the cameras (a typical example is a planar crystal that appears as a needle if observed from certain views). This is the reason why we stored information about the microphysical retrievals both for individual cameras and for the aggregated triplet.

It must be noted that the method of^17^ was originally developed from a training set of data belonging to only 2 out of the 10 field campaigns we provide in MASCDB. This opens the possibility for future researchers to exploit the additional data and fine-tune the classification, for example by discriminating between different types of planar crystals as plates and dendrites.

#### Riming degree estimation

The estimation of the riming degree of the particles, also described in Praz *et al*.^17^, is provided in an analogous way both for individual cameras and aggregated for the triplet. The method discriminates between five discrete riming degree levels (unrimed, rimed, densely-rimed, graupel-like, graupel) and also provides a continuously varying riming degree *R*_*c*_ defined in the interval 0–1, with 1 being graupel. The relevant attributes in MASCDB start with *riming_*. Riming degree estimation presents some known limitations in the experience of the authors: small and bright particles may be classified as graupel and more in general their riming degree level can be unreliable. Similar caveats apply to out-of-focus images.

#### Melting snow identification

Liquid water droplets on the surface of snowflakes appear as very bright spots on the ROIs and therefore it is possible to try to identify melting snow visually. This is the third retrieval described in Praz *et al*.^17^. The continuous probability of melting in the interval 0–1 is provided as well as a discrete flag (attributes starting with *melting_*). The estimation is conducted, and separately stored, both on individual views (individual cameras) and as an average value of each triplet. When the probability of melting is larger than 0.5, the degree of riming is not anymore reliable and the riming degree information is set to *undefined* in the data.

Although we did not censor the output of hydrometeor classification, riming degree estimation and melting according to image quality *ξ*, we recommend to treat with care the estimates corresponding to *ξ* < 9 as they did not belong to the original training set used for algorithm development. For the sake of completeness, we also flagged with dedicated boolean attributes (attributes starting with *hl_*, meaning “human label”) the images which belonged to the visually-interpreted training set employed by Praz *et al*.^17^ in order to provide the readers with a benchmark to test eventual new and improved methods.

#### Mass, mass distribution and volume estimation

The recent 3DGAN (3-Dimensional Generative Adversarial Network) method described in Leinonen *et al*.^23^ showed some promising results concerning the estimation of mass and three-dimensional properties using as input a triplet of MASC images. 3DGAN is based on machine learning, using a large number of simulated snowflakes as training set. We provide, in the variables starting with *gan3d_* the estimation of total mass, volume and radius of gyration for the snowflakes for which 3DGAN could provide an estimate. 3DGAN requires good quality images in all the three views and it is not suited for particles classified as *small_particle* or particles undergoing melting and for this reason not all the triplets in MASCDB fulfill these requirements. Furthermore, some limitations of 3DGAN must be mentioned here. The simulated snowflakes used to train the algorithm included aggregates of various monomers and of different degrees of riming, but they were generated at a resolution of 40 *μ*m. This is comparable with the resolution of the MASC but not enough to fully depict the interaction of supercooled liquid water droplets of micron-size with the complex spatial structures of typical snowflakes. Additionally, 3D-GAN has been validated on a limited number of physical samples (3D printed snowflakes), also having a spatial resolution of 40 *μ*m.

#### Blowing snow identification

Blowing snow can be an unwanted source of noise in the data collected by the MASC or constitute relevant information in itself^30^. During blowing snow events, the MASC is triggered more often and many small ROIs may be recorded in the raw images of the three cameras. The method described in Schaer *et al*.^21^ employed image processing techniques on the raw MASC images and estimated if the population of particles recorded by the MASC instrument at a given time is more likely to belong to one of three classes: pure blowing snow, pure precipitation or precipitation mixed with blowing snow. In the last case, a mixing index ranging from 0 to 1 is also provided. We provide the predictions of this methodology with variables starting with *bs_*.

The availability of this estimate is useful for instance as a filter, when measurements from different field campaigns are merged together. A field campaign like *Davos-2015*, conducted in a wind protected environment, has only 0.3% of total triplets estimated to belong to a pure blowing snow population while for a field installation like *APRES3-2017* on the coasts of Antarctica where katabatic winds blow^31^ this proportion increases to 28%.

### Environmental data

It is relevant and may be useful to have access to basic environmental conditions in the proximity of the MASC instrument at the time of each image record. MASCDB provides (with attributes starting with *env_*) information about air temperature, relative humidity (with respect to water), wind speed, wind direction and atmospheric pressure. During all the campaigns this information was available in the close proximity (within 10 m) of the instrument, although from different sensors and different temporal resolutions ranging from seconds to minutes, as summarized in Table 2. Some data were collected by research instruments, temporarily deployed during selected field campaigns (using in all cases a Vaïsala WXT520 weather station) while in other cases we could access data collected by operational weather services. Data have been re-sampled at a common interval of one minute before being matched temporally to the closest MASC observation. We are confident that variables as temperature, humidity or pressure are comparable among different field installations, while wind direction and wind speed should be treated with care when data of more field campaigns are aggregated, as the local set-up of the instruments (local wind shielding, etc) may play a significant role, especially for data not collected following operational protocols.

**Table 2:** Characteristics of the native environmental data before their upsampling or downsampling to a common temporal resolution of one minute.

Campaign	Data source	Original resolution [s]
*Davos-2015*	Research	600
*APRES3-2016*	Operational (MeteoFrance)	60
*Valais-2016*	Research	10
*APRES3-2017*	Operational (MeteoFrance)	600–3600
*ICEPOP-2018*	Research	10
*PLATO-2019*	Research	60
*Davos-2019*	Research	600
*Jura-2019*	Operational (MeteoSwiss)	600
*POPE-2020*	Research	5
*ICEGENESIS-2021*	Operational (MeteoSwiss)	600

The column: *Data source* is used to differentiate between temporary research installations and long-term operational sites.

### Field campaigns

We provide data collected during 10 field campaigns from 2015 until 2021, as listed in Table 1. The campaigns have been conducted in different locations worldwide. Even though the exact geographical locations and other relevant information is readily available in the data themselves, it is worth to briefly describe the context and specificity of each installation.

#### *Davos-2015*: 46.8297 N, 9.8093 E, 2540 m amsl

This installation took place in an optimal setting: the MASC instrument was set up in a site belonging to SPICE^32^ (Solid Precipitation Intercomparison Experiment) within a double fence wind protection, thus particularly protected from wind-related biases^21,29^.

#### *APRES-2016* and *APRES3-2017*: -66.6628 N, 140.0014 E, 41 m amsl

During the international APRES3 program^30,33^ (Antarctic Precipitation: Remote Sensing From Surface and Space), a MASC instrument was deployed at the Antarctic French base *Dumont d’Urville*. Installed on the Antarctic coast, the MASC was often exposed to strong winds and consequently it frequently collected images of wind-blown snow. Given the remote location, the data collected here are particularly interesting and still largely unexploited. The second campaign in this location (*APRES3-2017*) includes unique data collected also during the months of Antarctic winter.

#### *Valais-2016*: 46.1222 N, 7.2122 E, 2370 m amsl

The instrument was installed in a ski resort in the Swiss Alps.

#### *ICEPOP-2018*: 37.6652 N, 128.6996 E, 789 m amsl

ICEPOP (International Collaborative Experiment for PyeongChang Olympic and Paralympics) was an international research effort conducted during the winter Olympics game in 2018 in Korea. The MASC instrument was installed, as for *Davos-2015*, within a double fence protection, in the measurement site of Mayhills^34^.

#### *PLATO-2019*: -68.5752 N, 77.9659 E, 10 m amsl

The measurements of this installation were collected in the Davis research station in Antarctica during a summer field campaign^35^. Only a limited number of triplets have been collected by the MASC instrument in this occasion.

#### *Davos-2019*: 46.8450 N, 9.8716 E, 1512 m amsl

MASC was installed in the Alps of eastern Switzerland in the context of Role of the research program: Aerosols and Clouds Enhanced by Topography on Snow (RACLETS)^36^). The installation site is not the same as in *Davos-2015*.

#### *Jura-2019*: 46.6702 N, 6.3125 E, 1045 m amsl

The instrument was deployed in western Switzerland, in the Jura mountains.

#### *POPE-2020*: -71.9499 N, 23.3471 E, 1382 m amsl

This installation took place in Antarctica, as a contribution to the Princess Elizabeth Antarctic Orographic Precipitation Experiment (POPE). The station was located in Queen Maud Land (East-Antarctica). Due to the presence of mountains of almost 3000 m of altitude in a 25 km radius from the station, the main goal of the campaign was to study the effects of the mountain range on clouds and snowfall at the site. The region, as well as the scientific base, are well described for example in Gorodetskaya *et al*.^37^.

#### *ICEGENESIS-2021*: 47.0830 N, 6.7922 E, 1018 m amsl

The instrument was installed in the Swiss Jura mountains in the framework of a field experiment contributing to the ICEGENESIS project (https://www.ice-genesis.eu/).

## Data Records

MASCDB can be accessed on Zenodo^28^. This section provides information about the data format and the data records. Any user should be able to read the data with the programming language of its choice. We recommend nevertheless to take advantage of the *pymascdb* package provided and described in the *Usage Notes* section. The dataset is composed of four *Apache parquet* (https://parquet.apache.org/, last accessed on Nov 1^*st*^ 2021) binary files containing scalar descriptors of individual snowflakes and one *Zarr* store where the gray-scale images of the corresponding ROIs are stored.

The four *Apache parquet* files are:

- MASCdb_triplet.parquet: records listed in Supplementary Table 3.
- MASCdb_CAM0.parquet: records listed in Supplementary Table 4.
- MASCdb_CAM1.parquet: records listed in Supplementary Table 4.
- MASCdb_CAM2.parquet: records listed in Supplementary Table 4.

The *MASCdb_triplet.parquet* file contains information that are valid for a triplet of images. For example, to cite a few entries, it includes environmental information, blowing snow detection, 3D-reconstruction parameters and MASC-estimated fall speed. The other three *Apache parquet* files (one for each camera) include the geometrical and textural descriptors as well as the retrievals computed over the ROIs identified in each camera view.

The *Zarr* store (named MASCdb.zarr) contains gray-scale images of all the triplets in the dataset. The four dimensional tensor with all triplet images has dimensions (number of triplets, y, x, camera id), shape (851697, 1024, 1024, 3) and is saved to disk in a *Zarr* array with chunk size (256, 1024, 1024, 3). The reason behind the choice of *Zarr*, with respect to other classical formats in use in the geoscience community for storing n-dimensional tensor, is that *HDF* and *netCDF*^38^ formats do not allow (without specialized installations) multi-threaded data input/output in python, introducing performance bottlenecks for the distributed computations enabled by the *pymascdb* introduced in the Usage Notes section. In order to work on common dimensions, every ROI of the triplet is centered into a grid of size 1024 × 1024 pixels by adding black (0) pixels around their boundary. Given a typical pixel size of 33.5 *μ*m, this correspond to a square box of about 3.5 cm side. Only one snowflake in the entire database exceeded the size of this bounding box of a few pixels; we decided therefore to keep the size of 1024 pixels, as it is a convenient power of 2, rather than further increase it.

## Technical Validation

### Snowflake geometry and microphysics

The availability of several geometrical and textural descriptors of the images, in combination with the output of retrieval algorithms tailored on snowflake microphysical characteristics, allows MASCDB users to perform all types of multivariate analysis.

**Fig. 5:**
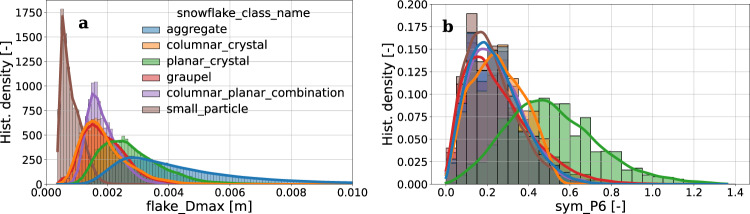
Conditional distribution (normalized histogram bin density and kernel density estimate KDE) of two geometrical descriptors stratified according to the estimated hydrometeor type. (**a**) Maximum dimension *D*_*max*_ (*flake_Dmax* attribute of Table 3). (**b**) Rotational symmetry attribute (*sym_P6* attribute of Supplementary Table 4). Data are filtered to remove all particles associated to blowing snow and *sym_P6* statistics are shown for CAM0.

For example, Fig. 5 stratifies the distribution of two geometrical descriptors according to the hydrometeor classification output. While it is reassuring but not surprising that aggregate snowflakes reach larger sizes with respect to other snowfall types, it is very interesting to observe the behavior of a more “complex” parameter: *sym_P6* (see Supplementary Table 4). The family of *sym_Pn* parameters are related to the *n*-fold symmetry of the images. The interesting signature is the increase of 6-fold symmetry for the hydrometeor category of planar crystals. Planar crystals include stellar and plate-like structures, as shown in Fig. 4 where the hexagonal basic structure of ice crystals is the most noticeable.

**Fig. 6:**
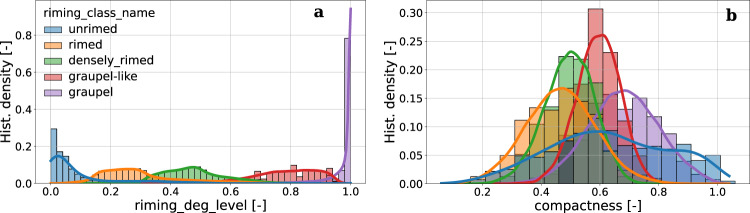
Conditional distribution (normalized histogram bin density and kernel density estimate KDE) of two snowflake attributes stratified according to the estimated riming degree class. (**a**) *riming_deg_level*. (**b**) *compactness*. All geometrical descriptors are listed and described in Supplementary Table 4. Data are shown for one of the three cameras (CAM0) and they are filtered to remove hydrometeors defined as *blowing_snow*, classified as *small_particles* and with riming degrees classified as *undefined*.

Data can be stratified also according to the riming degree level. Figure 6 provides the conditional distribution of the continuous riming degree index *R*_*c*_ (or *riming_deg_level*) with respect to the five discrete riming degree classes (left panel), as well as the distribution of a descriptor designed to capture particle compactness based on areal approximations with a fitted ellipse (*compactness*, right panel). While looking for example at *compactness*, a physically reasonable trend emerges: increasing riming degree levels are associated to more compact particles. The noteworthy exception is the large variability of *compactness* observed in particles classified as unrimed, so it is important to recall that: (i) compactness is only one among many descriptors used to depict the riming degree, (ii) the estimation of riming degree suffers of the limitations discussed previously, in case of small and bright particles.

### Environmental conditions and MASC measurements

The availability of co-located environmental measurements is a simple, important, and not very often available, source of information for microphysical investigations.

**Fig. 7:**
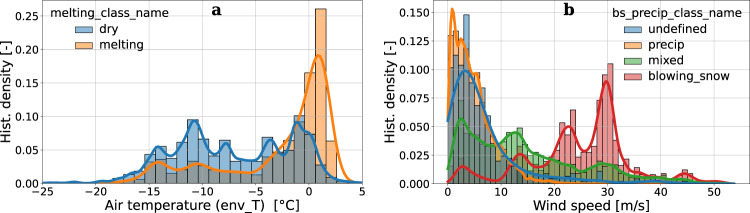
Conditional distribution (normalized histogram bin density and kernel density estimate KDE) of air temperature (**a**) and wind speed values (**b**). Temperature data are stratified according to the identification of melting particles (*melting_class_name* attribute in Supplementary Tables 3 and 4), while wind speed data are stratified according to the blowing snow detection classes (*bs_precip_class_name* attribute in Supplementary Table 3).

Figure 7 shows the MASCDB relationships between the identification of melting particles^17^ and air temperature, as well as the prediction of blowing snow occurrence^21^ and wind speed, while Fig. 9 presents the distribution of air temperature for aggregates larger than 5 mm. The results are physically-sound and they cross-validate the estimation methods which, in both cases, do not employ any environmental information for class predictions but use only the appearance of individual (or of a population of) particles. Particles labeled as *melting* are mostly measured around a temperature of 0 °C and there is a clear signature of particles associated to blowing snow when wind speed is very high. The large values of wind speeds must not surprise the reader, recalling that some field campaigns have been conducted in Antarctica where strong katabatic winds blow^31^. It is worth to underline, regarding the conditional distribution of air temperature with respect to melting particles, that a more appropriate environmental variable to consider for investigating this phenomenon would be the wet bulb temperature, which can be estimated by using relative humidity and atmospheric pressure data provided by MASCDB.

**Fig. 8:**
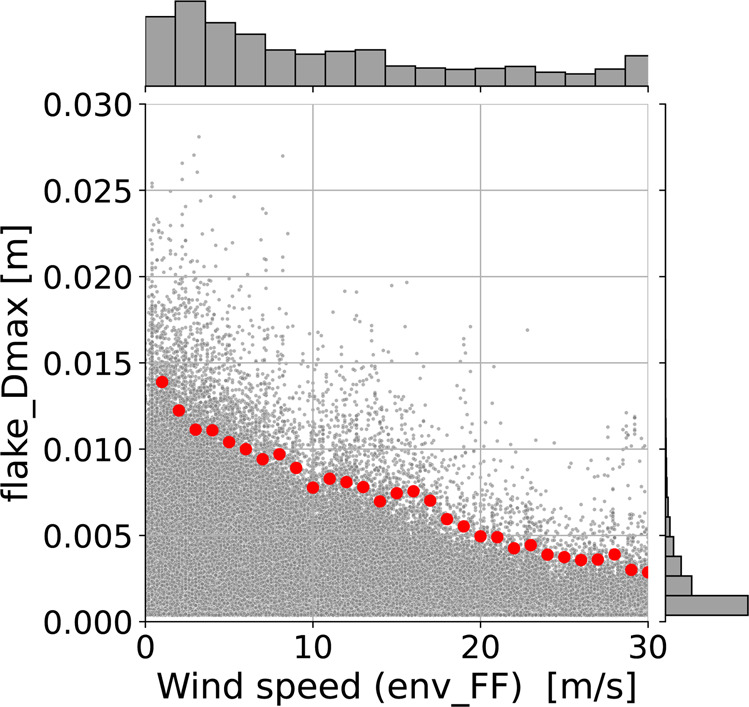
Scatterplot and histograms of wind speed values and maximum dimension *D*_*max*_ (*env_FF* and *flake_Dmax* attributes of Supplementary Table 3). The red dots highlight the 99% percentiles of *flake_Dmax* in bins of 1 ms^−1^ on *env_FF*.

Concerning the impact of winds on MASC measurements, previous studies have shown that wind speed affects the quality of hydrometeor fall speed data as recorded by the MASC^24^ and it reduces significantly the probability to observe particles of larger dimension^29^. A qualitative confirmation of this behavior of the MASC for the data in our database can be observed in Fig. 8: the larger the wind speed, the lower the maximum dimension of the observed particles.

### Parameterization of microphysical properties

MASCDB additionally paves the way for an enhanced understanding of the microphysical properties of frozen hydrometeors. This pertains to the field of weather and climate science, both through cloud and precipitation models and the microphysical parameterizations they rely on, and for the remote sensing community in the development of more realistic scattering models.

For instance, the mass estimates from 3D-reconstruction of MASC triplets^23^ allow to compute mass-dimensional relations, which are typically represented with power laws of the form $m=a{D}_{max}^{b}$. For each class of snow particle, each precipitation event in the database yields a pair (*a*, *b*), as shown in Fig. 10 for planar and columnar crystals, aggregates and graupel. These results show a relatively good agreement with results from previous studies^39–44^, in which (*a*, *b*) were derived from *in-situ* measurements. The largest discrepancy is seen for planar crystals; this could be related to the more challenging identification of this particle type, whose aspect can be quite different in the different camera views. An interesting outcome of this analysis is the strong correlation that is observed between exponent and prefactor values, which supports the findings of^45^, and which highlights that snow particle growth is driven by highly structured mechanisms. Additionally, it appears that while the different particle types have relatively similar *a*-*b* relations, the distribution of the exponent *b* varies significantly; in particular, graupel particles have a fractal dimension close to 3, consistent with their sphere-like geometry.

**Fig. 9:**
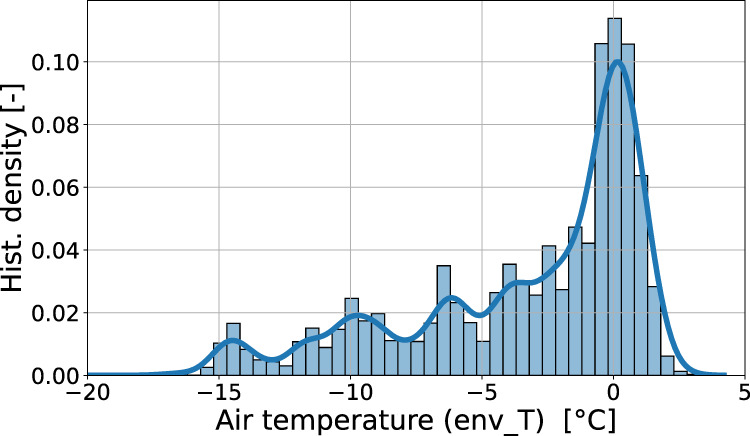
Distribution (normalized histogram bin density and kernel density estimate KDE) of air temperature value (*env_T* attribute of Supplementary Table 3) for particles of maximum dimension (*flake_Dmax*) larger than 5 mm and classified as aggregates. Data are filtered to remove *blowing_snow*.

**Fig. 10:**
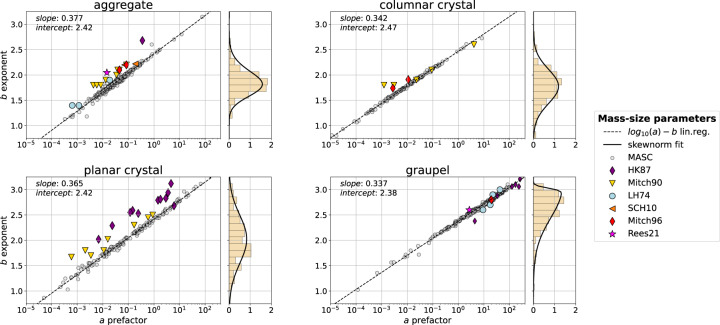
Mass-size coefficients (*a, b*) derived from snow particle distributions for all events in MASCDB (i.e. in the entire dataset) separated into particle types, along with literature values. MASCDB data are filtered to remove *blowing_snow*. Normalized histograms of the *b* exponent are displayed in the marginal plots. The literature references are: HK87^40^, Mitch90^41^, LH74^39^, SCH10^43^, Mitch96^42^, Rees21^44^.

Besides evidencing these features, this method provides distributions for *a* and *b* parameters, rather than point values as were typically computed from previous studies. This in turn allows for a statistical approach in the microphysical representation of frozen hydrometeors, which could additionally be fine-tuned according to riming degree, environmental conditions, campaign location, etc. Similar results are obtained when other properties are studied such as the area-size relation, as well as aspect ratio distribution, and potentially multiple other features.

### Perspectives on snowflakes representation learning, classification and generative modeling

As demonstrated by recent studies^18–20,23^, the usage of specialized deep learning architectures offers an interesting perspective for improvement and automation of MASC data information extraction. While supervised classification methods have been developed for this purpose in recent years^18,20^, their ability to generalize is limited by the representativeness of their labeled training datasets and the manual effort required for its assembly. Additionally, the definition of classes is subject to the scientist judgment and introduces artificial class boundaries which might not reflect the actual formation and continuous evolution of snowflakes shapes occurring in nature.

**Fig. 11:**
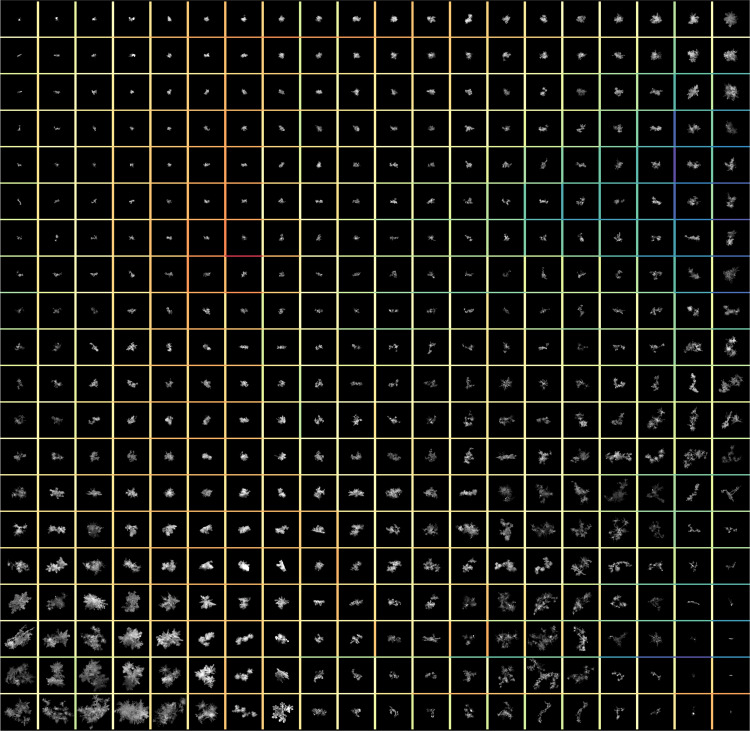
MASC sample for each self-organizing map cluster derived from image factors of variation of Leinonen *et al*.^19^. Neighbours images shares similar latent representations, with color borders encoding the average Euclidean distance between them (blue high similarity, red low similarity).

An alternative approach, which Leinonen *et al*.^19^ have started to explore, consists in the development of unsupervised techniques to divide a dataset into classes without expert-knowledge (training labels) provided by the scientists. Such methods rely on the ability of representation learning algorithms^46–54^ to recognize the important modes of variations in the images and encode their disentangled representation into a (latent) vector. Most of this continuous and discrete generative factors are usually constrained to be invariant to translation, rotation, and orientation of the image. Depending on the dimensionality of the latent space, these image descriptor factors can be further reduced in dimensionality^55–57^ to better investigate the encoded properties in low-dimensional manifolds^58–60^. Unsupervised classification can be performed by applying custom clustering algorithms on the latent factors^61–63^ or by including clustering specific loss into the neural networks^64–66^. Figure 11 illustrates the representational disentangling ability of such algorithms, by displaying MASC images randomly sampled for each cluster obtained by applying self-organizing maps^67^ to the factors of variations derived by the algorithm described in Leinonen *et al*.^19^.

The latent factors of a specific MASC image can be exploited also to research similar ones^68,69^: either by searching for existing MASC images whose latent factors lie closely to those of the anchor image, or alternatively by sampling from the latent space joint distribution and exploit the algorithm decoder/generator to simulate MASC images associated to the encoded latent structure. Such workflows would allow, for example, to investigate which environmental conditions are associated to similar snowflake structures, or alternatively to tentatively simulate the smooth evolution of a snowflakes conditional to some latent factor of variations by traversing the latent space.

Advances in generative adversarial networks^70–74^ have also improved the algorithmic capabilities for image enhancement^75^ and restoration^76–79^, which could be exploited to improve the quality of MASC images flagged to be of low quality (low *ξ*) and consequently improve the number of usable MASC images. Similarly, although algorithms for snowflake 3D reconstructions are already available^22,23^, it is foreseeable that advances in 3D modeling^80^ and representation learning^81^ will allow to further refine snowflake 3D reconstructions, microphysical retrievals and potentially scattering simulations.

## Usage Notes

MASCDB data are shared with the scientific community together with a python^82^ package named *pymascdb* which aims to facilitate data manipulation, analysis and visualization, besides providing utility methods for future database extension and algorithms development.

The *pymascdb* python package documentation, available at https://github.com/ltelab/pymascdb and https://pymascdb.readthedocs.io/en/latest/index.html, describes the usage of all the package functions and provides several tutorials to introduce the users to the data structure and the MASC_DB class methods.

When using *pymascdb*, all MASCDB data can be read in python into a MASC_DB class instance. The tabular data stored into the *Apache parquet* files are loaded in RAM memory into *pandas*^83^ objects, while the four-dimensional tensor with MASC images saved in the *Zarr* store is opened in “lazy mode” using *Xarray*^84^ with *Dask*^85^ as the array back-end. This means that the MASC images are not loaded into RAM, which allows to stream computation also on computing systems with not enough RAM to load the entire four-dimensional array into memory. Since operations on *Dask* arrays are lazy, operations such as filtering, sorting and image enhancement queue up as a series of sequential tasks mapped over blocks of data (*Dask* chunks). No computation is performed until the actual values of specific data chunks need to be computed (i.e. for visualization or image features extraction). At that point, data is loaded into memory and computation proceeds in a streaming fashion, block-by-block (*Dask* chunk by *Dask* chunk). More information can be found in the documentation of *Xarray* and *Dask* python packages.

Once a MASC_DB class instance is created, *pymascdb* enables the users to benefit from implemented class methods specifically designed to ease data manipulation operations such as data subsetting, filtering, sorting and reordering. For example, data can be filtered:

- by campaign (select_campaign, discard_campaign)
- by snowflake class (select_snowflake_class)
- by riming degree (select_riming_class)
- by blowing snow occurrence (select_precip_class)
- by apparent melting features (select_melting_class)

or reordered according to a specific image descriptors of a camera, for instance as:

mascdb.arrange (‘cam0.Dmax’, decreasing = True)

Many more examples are provided in the tutorials accompanying *pymascdb*.

To empower exploratory data analysis of MASCDB columnar format data (i.e. triplet, cam0, cam1, and cam2), *pymascdb* implements an accessor to the *Seaborn*^86^ data visualization library, to facilitate the visualization of multivariate relationships in multifaceted ways (see Figs. 5–9). Similarly, several plotting methods, built upon *Xarray* and *Matplotlib*^87^ libraries, are implemented to instantaneously display multiple MASC triplet images as well as camera-specific snowflakes. In order to improve the visual appearance of MASC images, *pymascdb* provides the possibility to correct the “raw” images with contrast stretching as well as global, adaptive and local histogram equalization algorithms. The plotting methods also allow to zoom close to the bounding box of the MASC image. For example, Fig. 4 was created with the *pymascdb* method plot_triplets, correcting the images with adaptive histogram equalization and zoom enabled.

To promote more detailed investigations, *pymascdb* also offers a utility to customize the definition of snowfall events. While the default *pymascdb* settings assumes a snowfall event to be defined by at least one MASC image acquisition within a time interval of 4 hours, the user can redefine the events by specifying parameters such as:

- maximum_interval_without_images
- event minimum_duration or
- event minimum_n_triplets.

While changing maximum_interval_without_images changes only the number of total events, the specification of the latter two parameters trigger a filtering of the dataset. After redefining snowfall events, the user can narrow down their selection of relevant events through implemented methods, for instance:

- select short events with mascdb.select_events_shortest(n = 20) or
- long-lasting snowfall mascdb.select_events_with_duration(min = np.timedelta64(2, ‘D’)).

MASCDB summary statistics of events and campaigns are also available typing mascdb.event and mascdb.campaign.

In conclusion, in the perspective of future inclusion of additional MASC images by upcoming campaigns and other institutions, *pymascdb* also implements tools to update or modify the MASCDB dataset presented in this manuscript, as well as it implements routines that might facilitate the development of new retrieval algorithms by the scientific community. For example,the method compute_2Dimage_descriptors enables the scientist to focus only on the development of a single custom-designed function that extract descriptors from a single image, and then *pymascdb* performs the heavy lift to scale the computation over all MASCDB images exploiting all the cores or cluster nodes available. The usage of a *Dask* array as *Xarray* back-end enables the user to choose between all available *Dask* schedulers which allows to perform multithreaded, multiprocess or distributed computing. If the *Dask* distributed scheduler is used (the current default), the image extraction computations can be profiled and monitored interactively in the *Dask Dashboard*.

A basic example of syntax is the following one, showing how Fig. 9 was generated, with all the filters applied to the data: #--------------------------------------------------------


# Import MASC_DB instance from api.py



from mascdb. api import MASC_DB



# Import plotting utils



import matplotlib as mpl



import matplotlib.pyplot as plt



# local path where all MASCDB files must be



dir_path = “/data/MASC_DB/”



# Create MASC_DB instance



mascdb = MASC_DB(dir_path = dir_path)



# Select only precip (discard mixed and blowing snow) and only aggregates



mascdb = mascd.select_snowflake_class(‘aggregate’). select_precip_class(‘precip’)



# Select only particles larger than 5 mm on Dmax



idx = mascdb.triplet[‘flake_Dmax’] >0.005



mascdb = mascdb.isel(idx)



# Plot taking advantage of seaborn integration



plt.figure(figsize = (10,6))



ax = mascdb.triplet.sns.histplot(x = ‘env_T’, kde = True, stat = ‘probability’, common_norm = False, binwidth = 0.5)



plt.show()


#------------------------------------------------------------

## Supplementary information

Supplementary Table 3

Supplementary Table 4

## Data Availability

The *pymascdb* package to manipulate the data and described in the previous section can be freely accessed at https://github.com/ltelab/pymascdb. Code documentation, examples and installation instructions are also available at https://pymascdb.readthedocs.io/en/latest/index.html.
