# Author Correction: MASCDB, a database of images, descriptors and microphysical properties of individual snowflakes in free fall

**DOI:** 10.1038/s41597-022-01375-6

**Published:** 2022-05-23

**Authors:** Jacopo Grazioli, Gionata Ghiggi, Anne-Claire Billault-Roux, Alexis Berne

**Affiliations:** Environmental Remote Sensing Laboratory, École Polytechnique Fédérale de Lausanne, Lausanne, Switzerland

**Keywords:** Hydrology, Cryospheric science, Hydrology, Cryospheric science

Correction to: *Scientific Data* 10.1038/s41597-022-01269-7, published online 03 May 2022

In this article the grant number 200020_175700/1 relating to Swiss National Science Foundation for Jacopo Grazioli was omitted. The original article has been corrected.

