# Supplementary material for: MASCDB, a database of images, descriptors and microphysical properties of individual snowflakes in free fall
Source: Sci Data. 2022 May 3;9:186. doi: 10.1038/s41597-022-01269-7 (PMC9065139; doi:10.1038/s41597-022-01269-7)
Supplement: Supplementary file 1 — Supplementary Table 3 [file 41597_2022_1269_MOESM1_ESM.pdf]

Parameter	Units	Type	Long name	Reference / Format / Algorithm
		Global information		
datetime		datetime		
campaign	-	string	Field campaign name	
latitude	deg	float	WGS84 latitude	
longitude	deg	float	WGS84 longitude	
altitude	m a m s l	float		
		Flake information		
flake_id	-	string	Unique flake id	*e.g. 2015.02.10_11.55.10_flake_4*<br>*YYYY.MM.DD_HH.mm.ss_flake_flake_number_tmp*
flake_number_tmp	-	string	Temporary flake id (not unique)	
flake_quality_xhi	-	float	Average quality index (on the three cams)	ξ in Praz et al, 2017[17]
flake_fallspeed	m/s	float	Recorded fallspeed	
flake_n_roi	-	int	Average # ROIs (on the three cams)	
flake_Dmax	m	float	Maximum Dmax (on the three cams)	Table A1:4 Praz et al, 2017[17]
		Riming estimation information		
riming_class_id	-	int	Discrete riming degree class id	Praz et al, 2017[17]<br>0: undefined, 1: unrimed, 2: rimed<br>3: densely-rimed, 4: graupel-like, 5: graupel
riming_class_name	-	string	Discrete riming degree class name	See riming_class_id
riming_class_prob	-	float	Riming classification probability	Praz et al, 2017[17]
riming_deg_level	-	float	Continuous riming degree level	R_c in Praz et al, 2017[17]
		Melting estimation information		
melting_class_id	-	int	Discrete melting class id	Praz et al, 2017[17]<br>0: dry, 1: melting
melting_class_name	-	string	Discrete melting class name	See melting_class_id
melting_prob	-	float	Melting probability	Praz et al, 2017[17]<br>If rounded, it yields melting_class_id
		Hydrometeor type estimation information		
snowflake_class_id	-	int	Hydrometeor class id	Praz et al, 2017[17]<br>1: small_particle, 2: columnar_crystal,<br>3: planar_crystal, 4: aggregate,<br>5: graupel, 6: columnar_planar_combination
snowflake_class_name	-	string	Hydrometeor class name	Praz et al, 2017[17]<br>See snowflake_class_id

Parameter	Units	Type	Long name	Reference / Format / Algorithm
snowflake_class_prob	-	float	Classification probability	
		3D reconstruction / mass estimation		
gan3d_mass	kg	float	Estimated mass	Leinonen et al, 2021[23]
gan3d_volume	m^3	float	Estimated volume	Leinonen et al, 2021[23]
gan3d_gyration	m	float	Estimated gyration radius	Leinonen et al, 2021[23]
		Co-located environmental information		
env_T	deg C	float	Air temperature	
env_P	hPa	float	Pressure	
env_DD	deg	float	Wind direction (North to East)	
env_FF	m/s	float	Wind speed	
env_RH	%	float	Relative humidity	
		Blowing snow estimation		
bs_precip_class_id	-	int	Blowing snow class id	Schaer et al. 2020[21]<br>0: undefined, 1: precip, 2: mixed, 3: blowing_snow
bs_precip_class_name	-	string	Blowing snow class name	Schaer et al. 2020[21]<br>See bs_precip_class_id
bs_normalized_angle	-	float	Blowing snow normalized angle	Schaer et al. 2020[21]<br>Pure precip if < 0.193<br>Pure blowing_snow if > 0.881
bs_mixing_ind	-	float	Blowing snow mixing index	Schaer et al. 2020[21]<br>Only defined in mixed BS/precip environments

Table 3. (**Supplementary**) Data records of the file *MASCdb_triplet.parquet*, containing descriptors and retrievals that are valid for the triplet of images as a whole.
