# Supplementary material for: MASCDB, a database of images, descriptors and microphysical properties of individual snowflakes in free fall
Source: Sci Data. 2022 May 3;9:186. doi: 10.1038/s41597-022-01269-7 (PMC9065139; doi:10.1038/s41597-022-01269-7)
Supplement: Supplementary file 2 — Supplementary Table 4 [file 41597_2022_1269_MOESM2_ESM.pdf]

Parameter	Units	Type	Long name	Reference / Format / Algorithm
**Global information**				
datetime		datetime		
campaign	-	string	Field campaign name	
pix_size	m	float	Pixel size / resolution	
n_roi	-	int	# of ROIs in raw image	Praz et al, 2017[17]<br>Note: only one main ROI<br>is considered in the descriptors
cam_id	-	int	Cam id: 0, 1, 2	
quality_xhi	-	float	Image quality index<br>(on the three cams)	ξ in Praz et al, 2017[17]
roi_width	pixels	int	x-size of the crop containing<br>the selected ROI	
roi_height	pixels	int	y-size of the crop containing<br>the selected ROI	
roi_centroid_X	pixels	int	X coordinate of ROI centroid<br>(with respect to original image)	
roi_centroid_Y	pixels	int	Y coordinate of ROI centroid<br>(with respect to original image)	
**Particle size and area**				
area	m^2	float	Particle area	Table A1:1 in Praz et al, 2017[17]
perim	m	float	Particle perimeter	Table A1:2 in Praz et al, 2017[17]
Dmean	m	float	Mean (x/y) dimension	Table A1:3 in Praz et al, 2017[17]
Dmax	m	float	Max dimension	Table A1:4 in Praz et al, 2017[17]
Dmax_ori	deg	float	Orientation of Dmax	
Dmax_90	m	float	Maximum dimension in the<br>orthogonal direction of Dmax.	
D90_r	-	float	Ratio of *Dmax_90* over *Dmax*	
eq_radius	m	float	Equivalent area radius	Table A1:5 in Praz et al, 2017[17]
area_porous	m^2	float	Porous area (holes removed)	Table A1:6 in Praz et al, 2017[17]
area_porous_r	m^2	float	Ratio area_porous over area	Table A1:7 in Praz et al, 2017[17]
**Elliptical approximation: fitted ellipse**				
ell_fit_A	m	float	Major semidimension	Table A1:8 in Praz et al, 2017[17]
ell_fit_B	m	float	Minor semidimension	Table A1:9 in Praz et al, 2017[17]
ell_fit_area	m^2	float	Area	Table A1:10 in Praz et al, 2017[17]
ell_fit_ori	deg	float	Orientation	Table A1:11 in Praz et al, 2017[17]
ell_fit_a_r	-	float	Axis ratio (ell_fit_A/ell_fit_B)	Table A1:12 in Praz et al, 2017[17]
ell_fit_ecc	-	float	Eccentricity	Table A1:13 in Praz et al, 2017[17]
compactness	-	float	area / ell_fit_area	Table A1:14 in Praz et al, 2017[17]
Elliptical approximation: inscribed ellipse (same center and orientation of fitted one)				
ell_in_A	m	float	Major semidimension	Table A1:15 in Praz et al, 2017[17]

Parameter	Units	Type	Long name	Reference / Format / Algorithm
ell_in_B	m	float	Minor semidimension	Table A1:16 in Praz et al, 2017[17]
ell_in_area	m^2	float	Area	Table A1:17 in Praz et al, 2017[17]
Elliptical approximation: circumscribed ellipse (same center and orientation of fitted one)				
ell_out_A	m	float	Major semidimension	Table A1:18 in Praz et al, 2017[17]
ell_out_B	m	float	Minor semidimension	Table A1:19 in Praz et al, 2017[17]
ell_out_area	m^2	float	Area	Table A1:20 in Praz et al, 2017[17]
Particle shape				
roundness	-	float	Area / circumscribed circle area	Table A1:30 in Praz et al, 2017[17]
p_circ_out_r	-	float	Perimeter / circumbscribed circle perimeter	Table A1:31 in Praz et al, 2017[17]
rectangularity	-	float	area / bounding box area	Table A1:32 in Praz et al, 2017[17]
bbox_width	m	float	Bounding box width	Table A1:33 in Praz et al, 2017[17]
bbox_len	m	float	Bounding box height	Table A1:34 in Praz et al, 2017[17]
rect_perim_ratio	-	float	Bounding box perimeter / perimeter	Table A1:35 in Praz et al, 2017[17]
rect_aspect_ratio	-	float	Bounding box aspect ratio	Table A1:36 in Praz et al, 2017[17]
rect_eccentricity	-	float	Bounding box eccentricity	Table A1:37 in Praz et al, 2017[17]
solidity	-	float	Area / convex hull area	Table A1:38 in Praz et al, 2017[17]
convexity	-	float	Convex hull perimeter / perimeter	Table A1:39 in Praz et al, 2017[17]
hull_n_angles	-	int	# of vertices of convex hull	Table A1:40 in Praz et al, 2017[17]
p_circ_r	-	float	Perimeter / equivalent area circle perimeter	Table A1:41 in Praz et al, 2017[17]
frac_dim_boxcounting	-	float	Fractal dimension boxcounting	Table A1:42 in Praz et al, 2017[17]
frac_dim_theoretical	-	float	Fractal dimension theoretical	Table A1:43 in Praz et al, 2017[17]
nb_holes	-	int	Number of holes inside the ROI	
Morphological skeleton				
skel_N_ends	-	int	Skeleton # of ending points	Table A1:44 in Praz et al, 2017[17]
skel_N_junc	-	int	Skeleton # of junctions	Table A1:45 in Praz et al, 2017[17]
skel_perim_ratio	-	float	Skeleton length / particle perimeter	Table A1:46 in Praz et al, 2017[17]
skel_area_ratio	m^{-1}	float	Skeleton length / particle area	Table A1:47 in Praz et al, 2017[17]
Rotational symmetry				
sym_P1 ... sym_P6	-	float	Standardized distance to centroid Fourier power spectrum component P1 to P6	Table A1:49-54 in Praz et al, 2017[17]
sym_Pmax_id	-	int	id of maximum value (sym_P∗)	Table A1:55 in Praz et al, 2017[17]
sym_P6_max_ratio	-	float	sym_P6 / max(sym_P∗)	Table A1:56 in Praz et al, 2017[17]
sym_mean	pixels	float	Mean distance to centroid	Table A1:57 in Praz et al, 2017[17]

Parameter	**Units**	**Type**	**Long name**	**Reference / Format / Algorithm**
sym_std	pixels	float	Standard deviation of distance to centroid	Table A1:58 in Praz et al, 2017[17]
sym_std_mean_ratio	-	float	sym_std / sym_mean	Table A1:59 in Praz et al, 2017[17]
			Texture operators (for ROI/particle)	
intensity_mean	-	float	Mean pixel brightness	Table A1:60 in Praz et al, 2017[17]
intensity_max	-	float	Maximum pixel brightness	Table A1:61 in Praz et al, 2017[17]
contrast	-	float	Contrast	Table A1:62 in Praz et al, 2017[17]
intensity_std	-	float	Pixel brightness standard deviation	Table A1:63 in Praz et al, 2017[17]
hist_entropy	-	float	Brightness histogram entropy	Table A1:64 in Praz et al, 2017[17]
local_std	-	float	Average local (3x3) intensity standard deviation	Table A1:65 in Praz et al, 2017[17]
local_intens	-	float	Average local (3x3) mean intensity	Table A1:66 in Praz et al, 2017[17]
lap_energy	-	float	Energy of the laplacian	Table A1:67 in Praz et al, 2017[17]
wavs	-	float	Sum of wavelet coefficients	Table A1:68 in Praz et al, 2017[17]
complexity	-	float	Particle complexity	Table A1:69 in Praz et al, 2017[17]<br>Garrett and Yuter, 2014[3]
			Haralick features	
har_energy	-	float	Haralick energy	Table A1:70 in Praz et al, 2017[17]
har_contrast	-	float	Haralick contrast	Table A1:71 in Praz et al, 2017[17]
har_corr	-	float	Haralick correlation	Table A1:72 in Praz et al, 2017[17]
har_hom	-	float	Haralick homogeneity	Table A1:73 in Praz et al, 2017[17]
			Riming estimation information	
riming_class_id	-	int	Discrete riming degree class id	Praz et al, 2017[17]<br>0: undefined, 1: unrimed, 2: rimed<br>3: densely-rimed, 4: graupel-like, 5: graupel
riming_class_name	-	string	Discrete riming degree class name	See riming_class_id
riming_class_prob	-	float	Discrete riming degree classification probability	Praz et al, 2017[17]
riming_deg_level	-	float	Continuous riming degree level	R_c in Praz et al, 2017[17]
			Melting estimation information	
melting_class_id	-	int	Discrete melting class id	Praz et al, 2017[17]<br>0: dry, 1: melting
melting_class_name	-	string	Discrete melting class name	See melting_class_id
melting_prob	-	float	Melting probability	Praz et al, 2017[17]<br>If rounded, it yields melting_class_id
			Hydrometeor type estimation information	
snowflake_class_id	-	int	Hydrometeor class id	Praz et al, 2017[17]<br>1: small_particle, 2: columnar_crystal,<br>3: planar_crystal, 4: aggregate,<br>5: graupel, 6: columnar_planar_combination

Parameter	Units	Type	Long name	Reference / Format / Algorithm
snowflake_class_name	-	string	Hydrometeor class name	Praz et al, 2017[17]<br>See snowflake_class_id
snowflake_class_prob	-	float	Classification probability	
**Human label information**				
hl_snowflake	-	int / bool	Human-label hydrometeor set	Boolean flag. If set to 1, this particle (in this CAM view) was part of the human labelled training set of Praz et al., 2017[17] for hydrometeor classification
hl_snowflake_class_id	-	int	Human-label hydrometeor class	Human-assigned ("true") *snowflake_class_id* of Praz et al., 2017[17]
hl_melting	-	int /bool	Human-label melting set	Boolean flag. If set to 1, this particle (in this CAM view) was part of the human labeled training set of Praz et al., 2017[17] for melting identification
hl_melting_class_id	-	int	Human-label melting class	Human-assigned ("true") *melting_class_id* of Praz et al., 2017[17]
hl_riming	-	int / bool	Human-label riming set	Boolean flag. If set to 1, this particle (in this CAM view) was part of the human labeled training set of Praz et al., 2017[17] for riming degree estimation
hl_riming_class_id	-	int	Human-label riming class	Human-assigned ("true") *riming_class_id* of Praz et al., 2017[17]

Table 4. **(Supplementary)** Data records of the three files *MASCdb_CAM<number>.parquet* (with number = *0, 1, 2*). The files contain information, retrievals, textural and geometrical descriptors of each camera view separately. Each file contain the same number of records in the same order.
